# Paradoxical contralateral hemiparesis in spontaneous spinal epidural hematoma: a case report

**DOI:** 10.1186/s12883-023-03179-6

**Published:** 2023-04-01

**Authors:** Kazuhiro Okada, Youshi Fujita, Ryuhei Kitai

**Affiliations:** 1 Department of General Medicine, Kaga Medical Center, Ri-36, Sakumi, Kaga-city, Ishikawa, 922-8522 Japan; 2 Department of Neurology, Fujita Neurological Hospital, 31-12-1, Hazaki, Maruoka, Sakai-city, Fukui, 910-0367 Japan; 3 Department of Neurosurgery, Kaga Medical Center, Ri-36, Sakumi, Kaga-city, Ishikawa 922-8522 Japan

**Keywords:** Hemiparesis, Paradoxical neurological sign, Spontaneous spinal epidural hematoma

## Abstract

**Background:**

Hemiparesis associated with spontaneous spinal epidural hematoma (SSEH) usually occurs ipsilateral to the hematoma. We here report the case of a patient with paradoxical hemiparesis contralateral to a spinal lesion due to SSEH.

**Case presentation:**

A 70-year-old woman was identified in routine clinical practice; she presented with acute-onset neck pain and left hemiparesis. Neurological examination showed left-sided sensory-motor hemiparesis without facial involvement. Cervical MRI showed a dorsolateral epidural hematoma compressing the spinal cord at the C2 to C3 level. Axial imaging demonstrated a crescent hematoma on the right side, which is contralateral to the hemiparesis, and lateral displacement of the spinal cord. Spinal angiography revealed no abnormal vessels. Based on clinical presentation and MRI findings, a diagnosis of SSEH was made. The patient was managed conservatively. The symptoms completely resolved without any neurological deficits, and the hematoma disappeared on the follow-up MRI.

**Conclusions:**

Paradoxical contralateral hemiparesis is one of the possible presenting symptoms in patients with SSEH. This case demonstrates the existence of the paradoxical contralateral hemiparesis associated with spinal compressive lesions. A plausible mechanism of the phenomenon is discussed.

## Background

Spontaneous spinal epidural hematoma (SSEH) typically presents with features ranging from simple neck or back pain to hemiparesis, complete paraplegia or quadriplegia, corresponding to the spinal levels and severity of cord or nerve root compression [1, 2]. Hemiparesis associated with SSEH usually occurs ipsilateral to the hematoma. Here, we report an atypical case of SSEH with motor and sensory deficits in the upper and lower limbs contralateral to the lesion.

## Case presentation

**Fig. 1:**
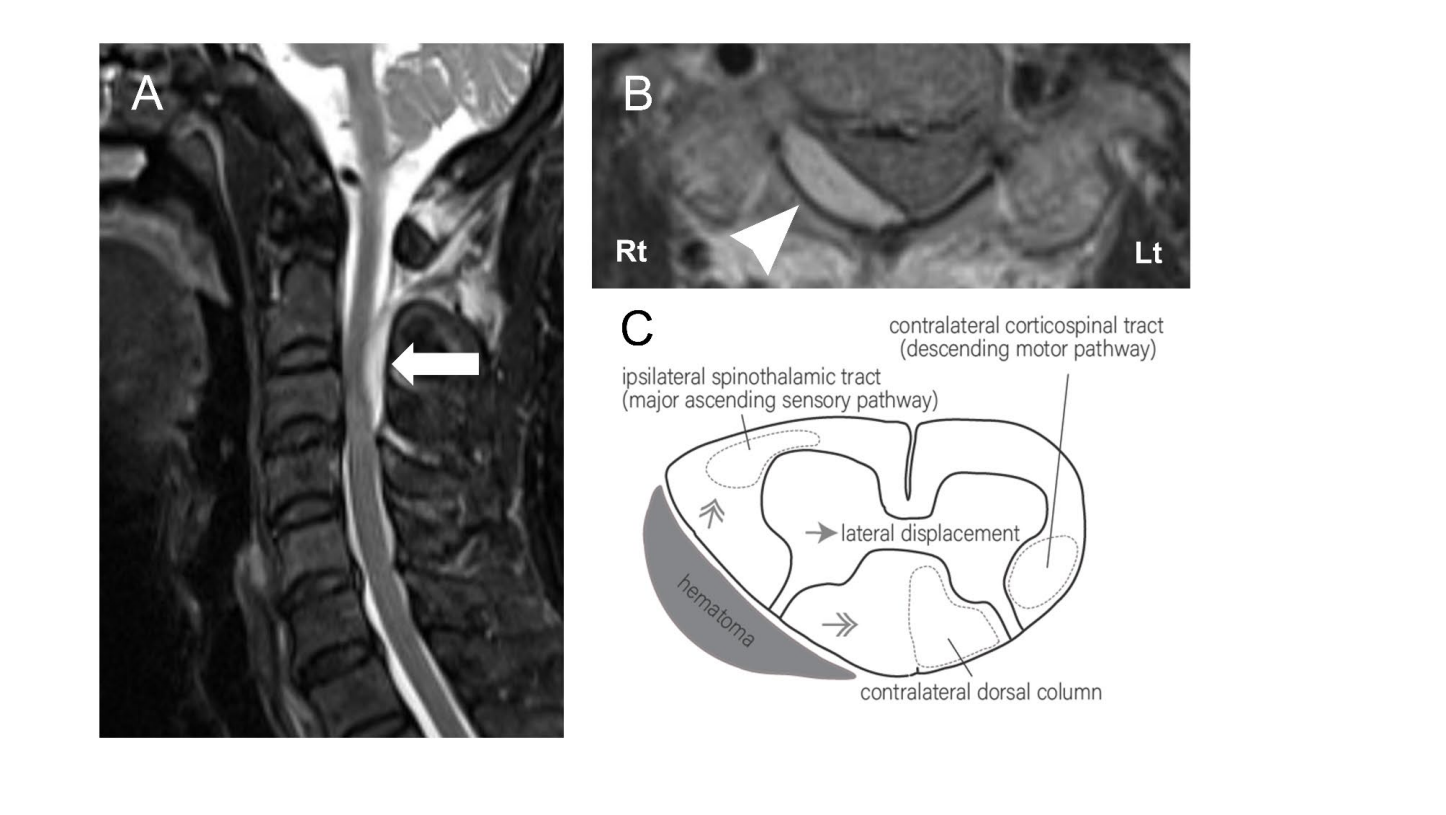
**A** Sagittal T2-weighted MRI shows a dorsolateral epidural hematoma compressing the spinal cord at the C2 to C3 level. **B** Axial FLAIR MRI shows a crescent hematoma on the right side and lateral displacement of the spinal cord. **C** Possible mechanism of paradoxical contralateral hemiparesis.

A 70-year-old woman presented to the emergency department with acute-onset neck pain and left hemiparesis. These symptoms occurred at rest without any specific trigger, and moving her neck exacerbated the pain. She had no history of recent trauma. Her medications included antihypertensive prescriptions and no antithrombotic agents. Her blood pressure on presentation was 230/129 mmHg. Physical examination revealed left-sided hemiparesis (manual muscle testing scores of 3/5 in upper and lower limbs), numbness and hypoesthesia on her left side, and no facial involvement. Laboratory studies showed no remarkable findings, including coagulopathy. Brain MRI and magnetic resonance angiography were performed to rule out stroke or vertebrobasilar artery dissection. Cervical MRI revealed an epidural hematoma localized to the dorsolateral spinal cord at the C2 to C3 level. Axial fluid-attenuated inversion recovery (FLAIR) imaging demonstrated a well-defined crescent hematoma on the right side, contralateral to the hemiparesis, and lateral displacement of the spinal cord (Fig. 1A, B). Spinal angiography revealed no abnormal vessels. Based on her clinical presentation and MRI findings, we diagnosed SSEH. Because of a remarkable improvement in her upper and lower limb symptoms within three hours after the onset, she was treated conservatively with a rigid cervical collar and antihypertensive agents. Her left-sided weakness and sensory deficits improved daily. Follow-up MRI on the sixth day showed that the hematoma had decreased in size. The hematoma had completely disappeared three weeks after the onset, the patient was fully recovered, and she was discharged without any neurological deficits.

## Discussion and conclusions

Among spinal disorders other than SSEH, hemiparesis contralateral to the spinal lesion has also been reported in cervical disc herniation [3] and lumbar synovial cysts [4]. These reports note the existence of hemiparesis contralateral to the spinal lesion.

Intracranial lesions, mostly those supratentorial and expanding, occasionally yield neurological signs not directly related to the location of the lesion. Hemiparesis ipsilateral to an intracranial lesion, referred to as false localizing signs or paradoxical hemiparesis, often led to wrong-site exploratory surgery in the pre-brain-imaging era [5]. Compelling candidate mechanisms of paradoxical hemiparesis due to intracranial lesions are (1) compression of the contralateral cerebral peduncle against the tentorial edge, referred to as Kernohan’s notch phenomenon; (2) vascular involvement of the contralateral hemisphere; or (3) impaired functional activation of the contralateral hemisphere [5].

In our case involving a spinal lesion, the mechanism of paradoxical contralateral hemiparesis may be analogous to Kernohan’s notch type injury, similar to that of a previously reported case with cervical disc herniation [3]. Pressure produced by the spinal hematoma could have been transferred across the spinal cord, and lateral displacement of the cord may have affected the contralateral corticospinal tract (descending motor pathway) and dorsal column, with direct compression of the ipsilateral spinothalamic tract (major ascending sensory pathway), causing the sensory-motor hemiparesis mimicking stroke (Fig. 1C).

We now note that contralateral hemiparesis is a possible presenting symptom in patients with SSEH. Our case suggests the rare occurrence of direct (ipsilateral) and indirect (contralateral) compression mechanism resulting in paradoxical hemiparesis, supplementing the few previous reports of similar contralateral hemiparesis associated with spinal compressive lesions.

## Data Availability

The data and images used in this case report are available from the corresponding author on request.

## Abbreviations

SSEH: Spontaneous spinal epidural hematoma
MRI: Magnetic resonance imaging
FLAIR: Fluid-attenuated inversion recovery.
